# Myocardial Contraction during the Diastolic Isovolumetric Period: Analysis of Longitudinal Strain by Means of Speckle Tracking Echocardiography

**DOI:** 10.3390/jcdd5030041

**Published:** 2018-08-09

**Authors:** Vicente Mora, Ildefonso Roldán, Elena Romero, Assumpció Saurí, Diana Romero, Jana Pérez-Gozalbo, Natalia Ugalde, Javier Bertolín, Melisa Rodriguez-Israel, Carmen Pérez-Olivares Delgado, Jorge A. Lowenstein

**Affiliations:** 1 Department of Cardiology, Hospital Dr Peset, 46017 Valencia, Spain; il.roldant@comv.es (I.R.); e.romero.dorta@gmail.com (E.R.); susi.sauri@gmail.com (A.S.); jana.castellon@gmail.com (J.P.-G.); bertolinbo@gmail.com (J.B.); carmenperezolivares@gmail.com (C.P.-O.D.); 2 Cardiodiagnosis Department, Medical Research of Buenos Aires, CP 1425 Buenos Aires, Argentina; dirzcardio@gmail.com (D.R.); natyuga@hotmail.com (N.U.); meli.1486@hotmail.com (M.R.-I.); lowensteinjorge@hotmail.com (J.A.L.)

**Keywords:** helical ventricular myocardial band, diastolic contraction, longitudinal strain, ventricular torsion, speckle-tracking echocardiography

## Abstract

Background: According to the ventricular myocardial band model, the diastolic isovolumetric period is a contraction phenomenon. Our objective was to employ speckle-tracking echocardiography (STE) to analyze myocardial deformation of the left ventricle (LV) and to confirm if it supports the myocardial band model. Methods: This was a prospective observational study in which 90 healthy volunteers were recruited. We evaluated different types of postsystolic shortening (PSS) from an LV longitudinal strain study. Duration of latest deformation (LD) was calculated as the time from the start of the QRS complex of the ECG to the latest longitudinal deformation peak in the 18 segments of the LV. Results: The mean age of our subjects was 50.3 ± 11.1 years. PSS was observed in 48.4% of the 1620 LV segments studied (19.8%, 13.5%, and 15.1% in the basal, medial, and apical regions, respectively). PSS was more frequent in the basal, medial septal, and apical anteroseptal segments (>50%). LD peaked in the interventricular septum and in the basal segments of the LV. Conclusions: The pattern of PSS and LD revealed by STE suggests there is contraction in the postsystolic phase of the cardiac cycle. The anatomical location of the segments in which this contraction is most frequently observed corresponds to the main path of the ascending component of the myocardial band. This contraction can be attributed to the protodiastolic untwisting of the LV.

## 1. Introduction

Left ventricular (LV) function relies on the contraction and relaxation of a complex myocardial fiber architecture organized as a syncytium, which determines changes in the shape and size of the LV [1,2].

One of the most widespread approaches to describe cardiac function and architecture is Torrent-Guasp’s ventricular myocardial band [3,4]. According to this model [5,6,7,8,9,10], the ventricular myocardium is a single muscular band composed of two loops: a basal transverse loop covering the right ventricle and the mid-basal portion of the LV, and an oblique loop in the form of a spiral with a descending and an ascending component wrapped around the LV. The transverse fibers of the basal loop enclose the upper two-thirds of the apical loop, while the ascending apical loop is located at the epicardium level and runs along the septal wall to the aortic root. Some fibers of the ascending component cover the free wall of the right ventricle and reach the base of the LV after crossing the posterior interventricular groove. These fibers, named “aberrant fibers” by Torrent-Guasp, are inserted throughout the course of the ventricular base, thus enveloping both ventricles [3,11]. The basal loop extends longitudinally in a spiral form from the base to the apex, with varying degrees of obliquity in relation to the subendocardium, and progressively wrapping around the ventricular cavity towards the apex and subepicardium, thus forming the descending component of the apical loop. The descending portion’s apical twist marks the beginning of the ascending component, which runs along the subepicardium with varying degrees of obliquity from apex to base, in the opposite direction to the subendocardial fibers.

Ventricular ejection is produced by myocardial contraction of the basal loop and the descending apical segment after stimulation of the endocardial Purkinje fibers [12,13], which occurs from the endocardium to the epicardium and from the apex towards the base. This characteristic transmural mechanical activation occurs as a result of the poor penetration of Purkinje fibers in the human myocardium [14] and anisotropic intramyocardial conduction [12,13,14,15].

According to the myocardial band model, the initial diastolic phase that follows ejection is a result of the muscular contraction of the ascending segment of the apexian loop [9,16,17], rather than passive isovolumetric relaxation. This theoretical anatomical–physiological behavior needs experimental confirmation.

Two-dimensional echocardiography allows us to analyze time and space in the complex mechanism of myocardial contraction and deformation in a noninvasive way. We examined segmental myocardial deformation of the LV by speckle-tracking echocardiography (STE), particularly during the initial diastolic isovolumetric period, and assessed its possible correspondence with the active contraction of the ascending segment of the apexian loop suggested by the myocardial band model.

## 2. Materials and Methods

### 2.1. Study Population

This was an observational, prospective study initially including 104 healthy volunteers randomly selected among hospital staff. Inclusion criteria were: age >18 years, absence of cardiovascular disease, and normal physical and electrocardiographic examination results. Exclusion criteria were sports training, pregnancy, and presence of cardiovascular risk factors.

The study was approved by the Clinical Research and Ethics Committee of the Hospital. The written informed consent of all the participants was obtained.

### 2.2. Echocardiography

We employed an ultrasound system (Vivid E9 and Vivid E95, GE Healthcare Medical Systems, Norway) equipped with a 2.5-MHz transducer.

Two-dimensional projections of the apical plane (four and two chambers, and long axis views) were obtained to calculate longitudinal strain (LS). All images were obtained with a frequency of 50–80 frames/s. The moment of aortic valvular closure was determined in the long axis apical projection. All examinations were transferred to a workstation for computer analysis (EchoPAC GE Healthcare software version 112.0.0).

To calculate LS, the LV endocardial border was traced manually. Next, a larger second concentric circle was automatically generated near the epicardium to include the entire thickness of the myocardium. The program divided each projection into six equal segments and performed speckle tracking frame by frame, providing automatized tracking confirmation (verified by the operator) and generating strain values, expressed as percentages. 

**Figure 1:**
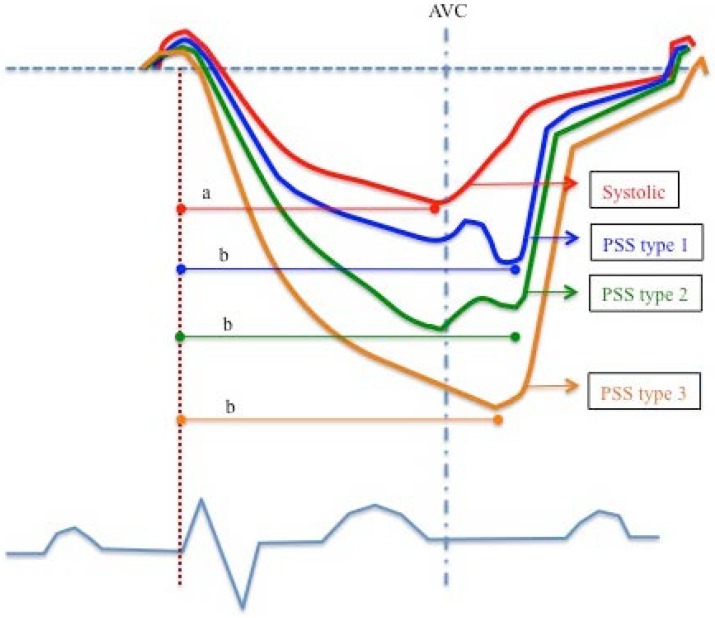
Morphological types of longitudinal deformation observed in healthy subjects. a: Duration of systolic deformation; b: Duration of postsystolic deformation. AVC: Aortic valve closure. PSS: Postsystolic shortening. (Referred only to the morphology, regardless of its amplitude).

**Figure 2:**
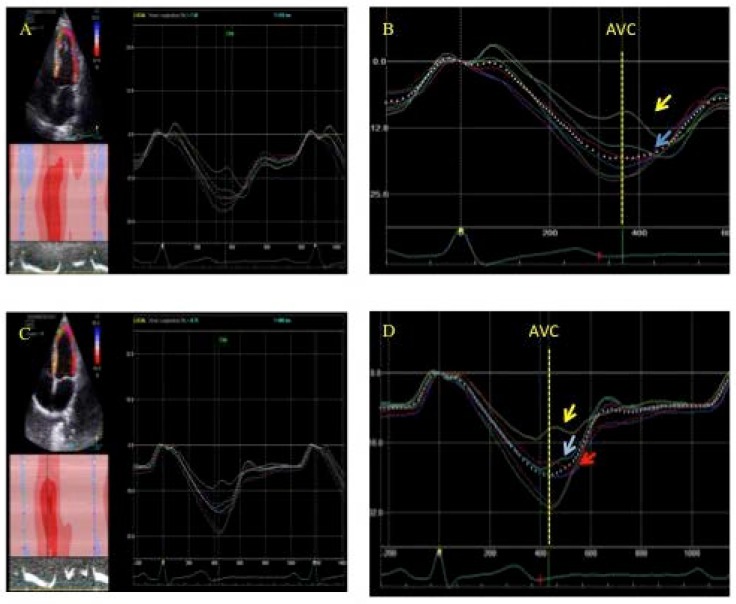
Postsystolic shortening (PSS) (**A**). PSS of the medial (light blue) and basal (yellow) segments of the interventricular septum, indicated by arrows in (**B**) (enlarged) (**C**). PSS of the medial (light blue) and basal (yellow) segments of the interventricular septum, and lateral basal (red) segment, indicated by arrows in (**D**) (enlarged). AVC: Aortic valve closure.

LS was used to analyze the occurrence of postsystolic shortening (PSS) (Figure 1 and Figure 2) and is defined as the presence of negative deflection after aortic valve closure. We could observe three types of PSS. Type 1: greater than the systolic deformation; Type 2: less pronounced than the systolic deformation; Type 3: the only deformation observed.

The duration of latest deformation (LD) was defined as the time from the beginning of the QRS complex in the ECG to the latest longitudinal deformation peak (Figure 1), be it systolic or postsystolic, prior to the onset of myocardial lengthening that occurs when there is protodiastolic filling.

We drew the bull’s eye map with the percentages of PSS registered for each of the 18 segments of the LV. The mean of LD times in each segment was used to estimate the segmental deformation sequence.

### 2.3. Statistical Analysis

Continuous variables are expressed as mean and standard deviation (SD). Strain values between men and women and between regions were compared using a Student’s *t* test as appropriate. A *p* value < 0.05 was considered statistically significant. 

The intraclass correlation coefficient was used to evaluate intraobserver and interobserver reproducibility of continuous variables for a random sample of 10 cases, with masking and measurements performed at different moments. Graphical representation was analyzed using the Bland–Altman method. 

Statistical analyses were performed using the IBM SPSS Statistics v.19.0.0329 software package.

## 3. Results

The study population finally consisted of 90 healthy subjects after excluding 14 due to a deficient ultrasonic window that made it impossible to obtain adequate measurements in all segments of the LV. 

Table 1 provides our study population’s characteristics. Women showed less body surface and ventricular volume, while no gender differences were detected in terms of ejection fraction. Intra and interobserver variability was good, with intraclass correlation coefficients of 0.86 (0.53–0.96; *p* < 0.001) and 0.87 (0.59–0.96; *p* < 0.001), respectively.

### 3.1. Postsystolic Shortening

PSS was observed in 48.4% of the 1620 segments analyzed (19.8% in the basal region, 13.5% in the medial region, and 15.1% in the apical region). PSS type 1 was the least frequent (9.4%), while PSS type 2 and 3 represented 19.6% and 19.4%, respectively. PSS types according to LV region are shown in Table 2. Type 3 was predominant in the apical region, while type 2 was more significant in the basal and medial zones.

**Figure 3:**
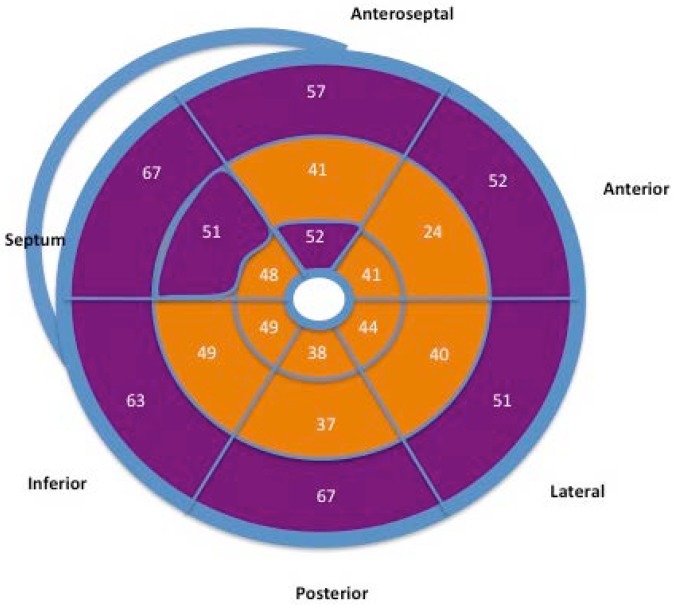
Bull’s eye map showing the frequency of PSS (%) in the different segments.

PSS percentage according to segment and type are shown in Table 3. Figure 3 shows a bull’s eye map of PSS distribution (percentage) according to segment. All LV segments presented PSS, but frequency was greater in the basal segments, medial septal segment, and apical anteroseptal segments (>50% of cases).

**Figure 4:**
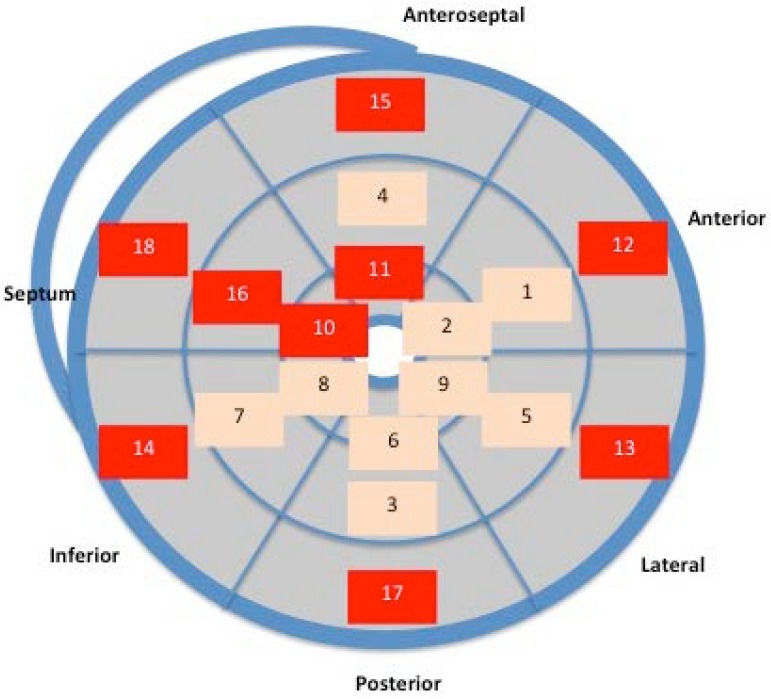
Segmental sequence of the mean time to longitudinal latest deformation (LD) in the 18 segments of the LV. The segments where LD occurs earliest are in yellow, and those where the peak of deformation occurs latest are in red.

The mean time to aortic valve closure was 372 ± 35 milliseconds. Table 4 shows the average time needed to reach LD in the 18 segments of the LV. The sequence is represented graphically in Figure 4, which shows how LD occurred earlier in the medial segments (except the septal) and in the anterolateral and posterior/inferior apical segments. LD took longest to occur in the interventricular septum segments, basal segments, and apical anteroseptal segment. The time difference in reaching LD between the first and last segment was 44 milliseconds. 

### 3.2. Anatomical Distribution

**Figure 5:**
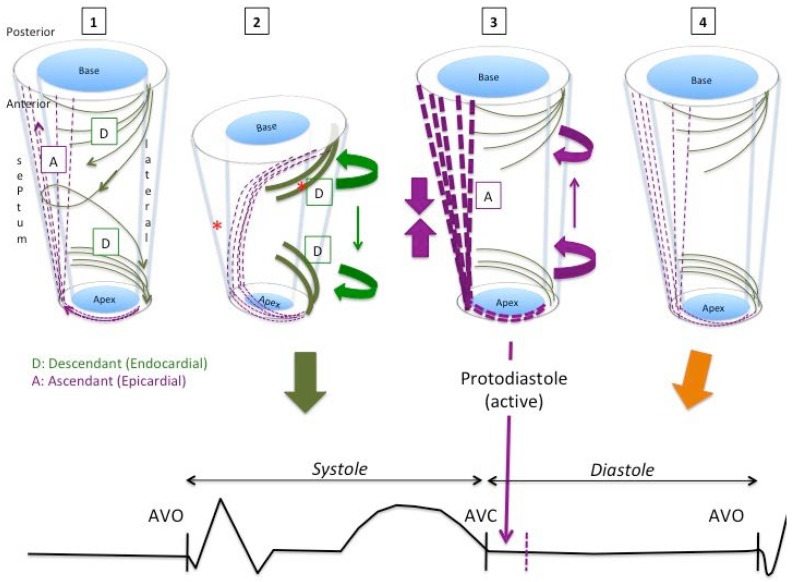
Frontal view. Diagram showing the behavior of the descending (green) and ascending (discontinuous purple) components of the apical loop. (**1**): In a telediastole, with the myocardium inactivated. (**2**): In a systole, electromechanical activation begins at the mid-septum and basal-lateral (asterisk), and spreads towards the apex and base, and from the endocardium to the epicardium. Contraction of the descending fibers (thick green) causes basal and apical rotation in opposite directions (twist) and longitudinal shortening of the ventricles by displacement of the base towards the apex, which results in stretching of the more epicardial ascending fibers (discontinuous purple). (**3**): The subsequent contraction of the ascending fibers (heavy discontinuous purple) causes longitudinal straightening and untwisting of the ventricle, coinciding with the period of isovolumetric filling. (**4**): Myocardial relaxation concludes during the diastole. NOTE: The basal loop, which wraps up and is stimulated simultaneously by the descending portion of the apical loop, is not represented in the graph.

**Figure 6:**
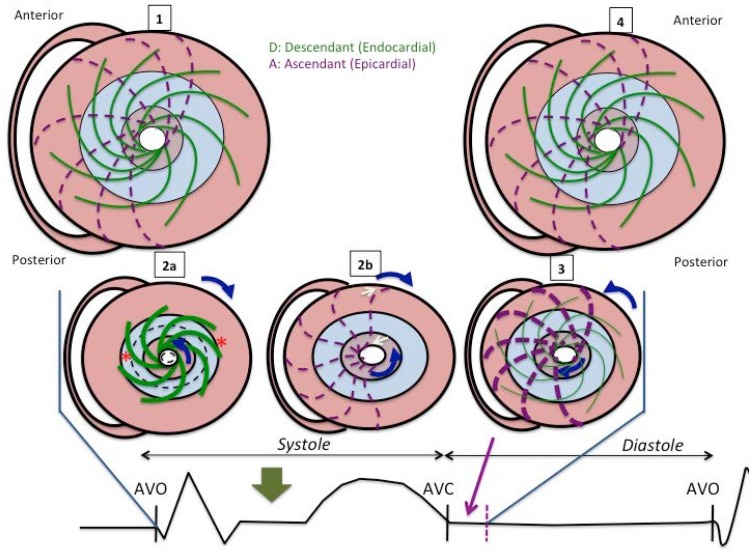
Diagram showing the behavior of the descending (green) and ascending (discontinuous purple) components of the apical loop viewed from the apex as a complement to the previous figure. (**1**): In a telediastole with the myocardium inactivated; (**2a**): The contraction of the descending fibers (thick green) causes basal and apical rotation in opposite directions (twist), and ventricular radial thickening; (**2b**): At the same time, the more epicardial ascending fibers (discontinuous purple) stretch at the ends (white arrows); (**3**): During the subsequent period of isovolumetric filling, the contraction of the ascending fibers (thick discontinuous purple) causes the longitudinal straightening and untwisting of the ventricle while relaxation of the descending fibers (thin green) takes place; (**4**): Myocardial relaxation concludes during the diastole.

In order to compare how our results were in line with the anatomical distribution of the apical loop described by Torrent-Guasp, we designed Figure 5 and Figure 6, which show myocardial behavior during the systole, isovolumic phase and diastole, and the corresponding actions on cardiac mechanics and their place in the cardiac cycle. 

Special emphasis is placed on the systolic stretching and postsystolic contraction of the ascending fibers (discontinuous purple lines) during their movement. The superposition of PSS distribution (Figure 3) and LD sequence (Figure 4) shows how the segments that make up the interventricular septum and the basal segments of the left ventricle were those with the highest percentage of PSS and where LD was most delayed. Moreover, they correspond to the main location of the ascending fibers described by the myocardial band model.

## 4. Discussion

We described the location of PSS and the temporal sequence of the left ventricle using the noninvasive technique of STE. The study population consisted of healthy subjects, so we could assume that PSS after aortic valve closure was the result of a contraction in the segment in which it was observed. 

PSS has been identified as a marker of myocardial dysfunction, mainly during cardiac ischemia and after infarction [18,19]. However, it is not pathognomonic for heart disease and has also been observed in healthy subjects [20], so its significance is somewhat unclear. The postsystolic contraction observed in patients with infarction is essentially different to the one observed in healthy patients due to its greater delay in relation to the aortic valvular closing [18,19,20].

STE provides information about the complex mechanism of myocardial contraction and the deformation that follows. In our study, PSS was observed in all of the segments of the LV, though the percentage was highest in the interventricular septum and the basal segments (Figure 3). Additionally, we were able to determine the LD sequence in each LV segment, highlighting the fact that it occurs later in the septal and basal segments (Figure 4).

PSS was described in healthy subjects by Voigt et al. [20], who used tissue Doppler echocardiography to detect PSS in 31% of LV myocardial segments, predominantly in the apical, basal anterior, and anteroseptal segments as well as segments of the septal wall. We could observe three types of PSS (Figure 1). At present, we do not know the intrinsic myocardial reasons of its distribution. Our data are in line with their findings, though we have observed a higher percentage of PSS in our series, probably because we obtained LS by means of STE (which is independent of ultrasonic orientation, unlike tissue Doppler) and took into account PSS type 3, detected mainly in apical segments. 

The localization of PSS and the LD sequence observed with STE provide functional data that endorse the muscle band theory of Torrent-Guasp [3,4,5,6,9]. According to this theory, the protodiastolic isovolumic phase of the cardiac cycle is a consequence of the active muscular contraction that occurs after aortic valve closure by the ascending portion of the apical loop. Its location corresponds to the anatomical distribution of the myocardial segments where PSS is detected and where LD occurs latest. Using simplified tractographic reconstruction with magnetic resonance imaging, Poveda et al. [21,22] demonstrated that the ventricular myocardium has a continuous helical structure from the pulmonary to the aortic artery ring. This helical disposition crosses the subendocardial space towards the apex, forming a double spiral that connects with the subepicardial space, adopting different oblique orientations around the ventricle. The distribution of ascending fibers reach all the LV segments, although they are mainly located in the interventricular septum and around the aortic annulus.

**Figure 7:**
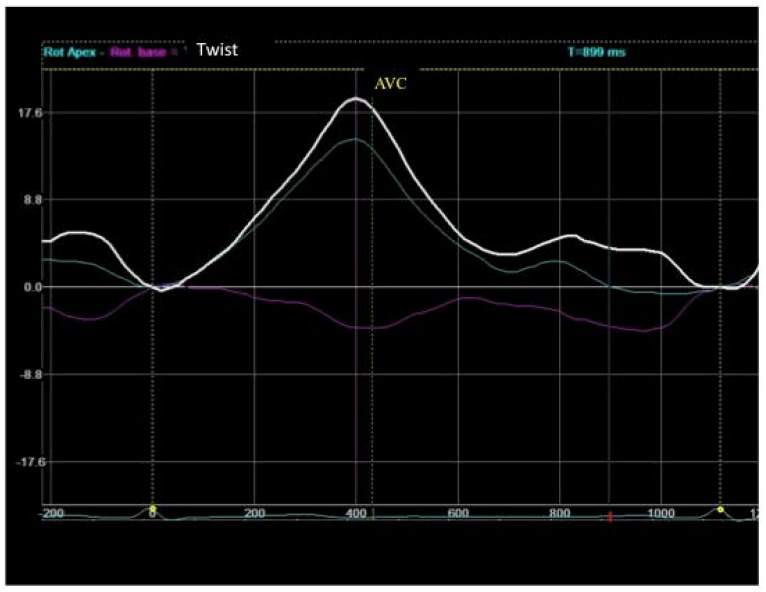
The simultaneous rotation in opposite directions of the base and the apex can be observed in the representation of the twist of a healthy adult subject obtained with EST.

According to the myocardial band model, blood is expelled as a result of the contraction of the basal loop and the descending segment of the apex loop. This contraction produces a thickening of the myocardium towards the ventricular centroid and “screws” the base to the apex by its simultaneous rotation in opposite directions (twist) (Figure 7), thereby “squeezing” the blood out as if it were a towel being wrung. 

While this occurs, the ascending portion of the apexian loop (with a more epicardial disposition), which mainly covers the descending segment at the septum up to the aortic root and the base of the LV, is “stretched” (Figure 5 and Figure 6).

Torrent-Guasp’s myocardial band model describes the initial diastolic phase following ejection as a phenomenon that arises due to the muscular contraction of the ascending segment of the apexian loop [9,16,17]. When ventricular blood ejection ends, the systole terminates and the diastole initiates with an isovolumetric period of “supposed relaxation”, during which intraventricular pressure decreases at a constant speed [23]. One of the key points of the theory is that the diastolic isovolumic phase is a phenomenon generated by delayed epicardial muscular contraction, which in turn produces longitudinal elongation of the ventricle that separates the base from the apex and ventricular untwisting [9,16,17]. Mechanical ventricular activation has been described during the diastole [13,24]. The final segment of the muscular band is the ascending component of the apexian loop that covers the descending segment and part of the basal loop, which is “stretched” during the initial ventricular contraction (Figure 5 and Figure 6). When it eventually contracts due to its more epicardial situation, the ascending segment “unscrews” the base from the apex, increasing the longitudinal axis of the heart and producing a suction similar to what happens inside a cylinder when it moves away from its plunger, a dynamic that has been observed in humans in studies with magnetic resonance [25].

This hypothesis implies a later mechanical activation at the epicardial level. Sengupta et al. [26] found the heterogeneity in segmental and transmural electromechanical activation in the anterior wall of the LV to be greater at the beginning of the diastole in a porcine experimental model. The delayed shortening of subepicardial fibers observed during the protodiastole correlates with the time required for the LV to reach the lowest protodiastolic pressure, suggesting it represents an active component of ventricular “relaxation”, which promotes suction early in the diastole. According to Buckberg [27], the contractile muscular force of the ascending segment causes elevation of the cardiac chamber due to the shortening (negative strain) registered during this phase. In our study, the parts of the LV in which the postsystolic negative strain, defined as PSS, was most frequent were the interventricular septum and basal segments. This can be interpreted as the origin of the active contractile movement, which leads to the untwisting and recovery of the longitudinal diameter after aortic valve closure. Figure 5 shows the paradoxical increase in the LV longitudinal diameter mediated by the myocardial contraction (negative longitudinal strain) of ascending fibers.

Fonafagou et al. [28] used electromechanic wave imaging to observe how LV electromechanical activation begins in the endocardium of the middle segments of the interventricular septum, anterior and posterior wall, and in the lateral wall endocardium near the base. These results were supported by Gurev et al. [29], who showed that both electrical and mechanical activation move from endocardium to epicardium and from apex to base. In fact, there is a transmural difference in the electromechanical delay through the LV free wall, which increases from apex to base. The poor myocardial penetration of the Purkinje network in humans plays a role here; from the moment the electrical impulse reaches the subendocardium, the electromechanical activation is transmitted anisotropically through the myocardial tissue [13,14,15]. In a report by Gurev et al. [29], basal and interventricular septum segments of the LV exhibited the longest electromechanical delay, which is anatomically consistent with the delayed LD we have observed in the temporal segmental sequence with STE.

Ashikaga et al. and Sengupta et al. described something similar with models of electrical activation, also observing that the LV subendocardium was activated before the subepicardium and the apex before the base [13,26]. In addition, the delay between electrical activation and initiation of mechanical shortening (electromechanical delay) was greater in the basal region than in the apical region. In the present study, we provided further information by determining the LD sequence using the noninvasive method of STE. As seen in Figure 6, the regional sequence of LD in the anteroseptal segments is in accordance with that of the LS at the anterior wall described by Sengupta et al. [26]. Using sonomicrometry, they observed that at 80% maximum deformation, the medium segments were the first in which LS appeared, followed by the apical and basal segments. 

Not only does the muscular component itself intervene in this process. The restoration of the potential energy that is generated when the myocardial tissue becomes deformed tends to return the myocardium to its resting form. The systolic deformation of the extracellular matrix [30] and myocyte components, such as elastic proteins and titin, generate a potential energy analogous to the compression of a spring. This potential energy (“recoil” or “restoration force”) has also been associated with rapid protodiastolic untwisting [31].

## 5. Conclusions

The presence of PSS observed with STE in myocardial segments that have initiated the relaxation process suggests the existence of an additional delayed active contraction after aortic valve closure. The anatomical arrangement of the segments in which PSS is most frequently recorded is consistent with the main path of the ascending component described by the myocardial band model of Torrent-Guasp. The postsystolic contraction of this component, which occurs later due to its epicardial situation, suggests it plays a role in the protodiastolic longitudinal elongation and untwisting of the LV and promotes the diastolic suction of the blood into the ventricular cavity. 

## Figures and Tables

**Table 1:** Characteristics of the control population (*n* = 90), differentiated by sex.

	Total (*n* = 90)	Men (*n* = 52)	Women (*n* = 38)	*p*
Age	50.3 ± 11.1	50.4 ± 11.1	50.1 ± 11.1	0.88
Body surface	1.8 ± 0.2	1.9 ± 0.1	1.6 ± 0.1	0.001
Heart rate	65 ± 10	65 ± 11	66 ± 9	0.65
Systolic blood pressure (mmHg)	119 ± 16	121 ± 16	115 ± 15	0.07
Diastolic left ventricle (mm)	45.5 ± 4.5	47.4 ± 4.2	43.0 ± 3.4	0.001
Left ventricular mass	151.6 ± 51.0	170.7 ± 53.6	125.4 ± 32.8	0.001
LVED (mL)	89.2 ± 28.4	101.1 ± 27.9	73.0 ± 20.1	0.001
LVES (mL)	29.8 ± 11.1	34.1 ± 11.5	24.1 ± 7.5	0.001
EFLV (%)	66.6 ± 5.5	66.4 ± 5.4	66.9 ± 5.7	0.68
Global Longitudinal Strain	−21.1 ± 2.1	−20.7 ± 2.0	−21.7 ± 2.1	0.02

LVED: Left ventricular end-diastolic volume; LVES: Left ventricular systolic volume; EFLV: Ejection fraction of the left ventricle.

**Table 2:** PSS and its types according to regions of the left ventricle (LV) (90 patients).

Segments	PSS Total	Type 1	Type 2	Type 3
Basal (*n* = 540)	321 (59.4%)	84 (15.5%)	139 (25.7%)	91 (16.8%)
Medial (*n* = 540)	218 (40.3%)	43 (7.9%)	102 (18.9%)	73 (13.5%)
Apical (*n* = 540)	245 (45.4%)	19 (3.5%)	76 (14.1%)	150 (27.8%)

PSS: Postsystolic shortening.

**Table 3:** PSS and its types according to segments of the LV (90 patients).

Segment	PSS (%)	Type 1 (%)	Type 2 (%)	Type 3 (%)
1 Basal anteroseptal	51 (56.6)	13 (14.4)	28 (31.2)	9 (10.0)
2 Basal septal	60 (66.6)	19 (21.2)	25(27.7)	16 (17.7)
3 Basal inferior	57 (63.3)	19 (21.2)	24 (26.6)	14 (15.5)
4 Basal posterior	60 (66.6)	9 (10.0)	29 (32.2)	22 (24.4)
5 Basal lateral	46 (51.1)	11 (12.3)	21 (23.3)	14 (15.5)
6 Basal anterior	47 (52.2)	19 (21.2)	12 (13.3)	16 (17.7)
7 Medial anteroseptal	37 (41.1)	6 (6.7)	17 (18.8)	14 (15.5)
8 Medial septal	46 (51.1)	7 (7.7)	28 (31.2)	11 (12.2)
9 Medial inferior	44 (48.8)	13 (14.4)	18 (20.0)	13 (14.4)
10 Medial posterior	33 (36.6)	6 (6.6)	16 (17.7)	11 (12.3)
11 Medial lateral	36 (40.0)	8 (8.9)	17 (18.8)	11 (12.2)
12 Medial anterior	22 (24.4)	3 (3.4)	6 (6.6)	13 (14.4)
13 Apical anteroseptal	47 (52.2)	2 (2.2)	18 (20.0)	27 (30.0)
14 Apical septal	43 (47.7)	2 (2.2)	16 (17.8)	25 (27.7)
15 Apical inferior	44 (48.8)	7 (7.7)	9 (10.0)	28 (31.1)
16 Apical posterior	34 (37.7)	2 (2.2)	16 (17.8)	16 (17.7)
17 Apical lateral	40 (44.4)	3 (3.3)	12 (13.3)	25 (27.8)
18 Apical anterior	37 (41.1)	3 (3.3)	5 (5.6)	29 (32.2)

PSS: Postsystolic shortening.

**Table 4:** Segmental sequence in ascending order: time to the latest longitudinal deformation (LD).

Segment	Mean (Milliseconds)	±SD
12 Medial anterior	372	43
18 Apical anterior	379	48
10 Medial posterior	382	47
7 Medial anteroseptal	385	51
11 Medial lateral	388	53
16 Apical posterior	389	57
9 Medial inferior	390	53
15 Basal lateral	391	51
17 Apical lateral	392	57
14 Apical inferior	394	58
13 Apical anteroseptal	395	55
6 Basal anterior	395	54
5 Apical septum	397	56
3 Basal inferior	405	59
1 Basal anteroseptal	405	60
8 Medial septum	407	74
4 Basal posterior	408	54
2 Basal septum	416	60
